# Iron–Carbon Nanospheres as Promising Material for Magnetic Assisted Adsorption and Separation of Impurities from a Liquid Phase

**DOI:** 10.3390/ma17092111

**Published:** 2024-04-29

**Authors:** Iwona Pełech, Sabina Lewinska, Monika Arciszewska, Abdul Khaliq, Anna Ślawska-Waniewska, Daniel Sibera, Piotr Staciwa, Urszula Narkiewicz

**Affiliations:** 1 Department of Inorganic Chemical Technology and Environment Engineering, Faculty of Chemical Technology and Engineering, West Pomeranian University of Technology in Szczecin, Pułaskiego 10, 70-322 Szczecin, Poland; daniel.sibera@zut.edu.pl (D.S.); piotr.staciwa@zut.edu.pl (P.S.); urszula.narkiewicz@zut.edu.pl (U.N.); 2 Institute of Physics, Polish Academy of Sciences, Aleja Lotników 32/46, 02-668 Warsaw, Poland; lewinska@ifpan.edu.pl (S.L.); arcis@ifpan.edu.pl (M.A.); akhaliq@ifpan.edu.pl (A.K.); slaws@ifpan.edu.pl (A.Ś.-W.); 3 Department of Construction and Road Engineering, Faculty of Civil and Environmental Engineering, West Pomeranian University of Technology in Szczecin, Piastów 50a, 70-311 Szczecin, Poland

**Keywords:** carbon spheres, iron, cementite, magnetic properties

## Abstract

The composites containing various iron compounds and highly microporous carbon spheres were produced and investigated for structural and magnetic properties. Iron citrate, nitrate and chloride were used to prepare samples and the obtained products contained iron, iron carbide or magnetite. All the produced samples were characterized by high porosity and good magnetic properties. The coupling of the high porosity of carbon spheres with magnetic properties of iron compounds provides a potential application of the composites to removal of impurities from water, followed by a magnetic separation of the sorbent.

## 1. Introduction

Various nanocarbon materials are known for their excellent adsorption properties. Among them, highly porous spherical carbon materials can be distinguished. Their spherical shape allows for better mechanical resistance and optimal package density in application. Spherical carbon materials have high specific surface area and porosity, resulting in excellent adsorption properties. They can be produced using high temperature or low temperature methods, including the Stöber’s method. The latter one was applied in a modified version by Liu et al. [1] who produced carbon spheres using a Teflon autoclave and carbonizing the samples under nitrogen. Our research group applied instead an autoclave a microwave assisted solvothermal reactor [2], which resulted in improving the product quality and significant shortening of the process run. Thanks to highly porous uniform structure the produced carbon spheres can be applied as sorbents of many species harmful for environment. When a porous sorbent is applied to removal of impurities in the liquid phase, an issue of the separation of the sorbent after the carrying out the adsorption process occurs. It can be solved by doping the carbon material with some magnetic species. Das et al. [3] presented such an approach for deposition of iron oxide nanoparticles on the 2D-nanosheets which rendered the entire material magnetically active. In the metal-carbon composite the high adsorption properties of the microporous carbon material are coupled with good magnetic properties of a decorating metal compound, enabling to separate the used sorbent applying a strong external magnetic field. For example, Pourzare et al. [4] described the application of graphene oxide/Co_3_O_4_ nanocomposites for the removal of organic dye pollutants from water, using magnetic field for the sorbent separation. Yang et al. [5] applied for the same purpose magnetic Fe_3_O_4_-activated carbon nanocomposite, and stated that such a system had demonstrated perfect magnetic separation performance and a high adsorption capacity of 321 mg/g for methylene blue from aqueous solution. The decrease of contamination of surface water is one of the crucial challenges recently. Water is contaminated with heavy metals [6], pesticides [7], pharmaceuticals [8] and dyes. The latter contaminants are particularly dangerous because of their toxic, mutagenic and carcinogenic properties [9]. The volume of “colored used water” released every year is estimated at the level of 200 billion L. Dye adsorption on solid sorbents (e.g., on carbon materials) is one of the methods of their removal. Moosavi et al. in their recent minireview [10] comprehensively described the use of a combination of a magnetic material and an activated carbon material for dye adsorption in wastewater treatment. Their literature survey underlines the evidence of the potential use of these magnetic adsorbents, as well as their magnetic separation and recovery. Taking into account such a potential application of iron-carbon composites to magnetically assisted adsorption and separation of impurities from liquid phase, we investigated structural and magnetic properties of the composites based on carbon spheres and iron compounds.

## 2. Materials and Methods

The composites containing carbon spheres and iron compounds derived from various precursors were produced. In all the samples the mass ratio of iron to carbon was as 1:10. Some samples were additionally activated with potassium oxalate or potassium hydroxide, keeping the mass ratio of carbon to potassium at 1:7. Various iron salts were applied as iron precursors: nitrate (Fe(NO_3_)_3_·9H_2_O), chloride (FeCl_3_·6H_2_O), or citrate (C_6_H_5_O_7_Fe). Formaldehyde and resorcinol were used as carbon precursors. To produce the composites, at first resorcinol and an iron precursor were dissolved in the water-ethanol mixture. Next, ammonia water (25%) was added to precipitate Fe(OH)_3_ and formaldehyde was dropped into the mixture. The whole mixture was placed in the microwave assisted solvothermal reactor (Ertec-Poland) and treated at 20 at for 15 min. In the case of activated samples potassium oxalate was added before solvothermal treatment and potassium hydroxide—after it. The last step of the preparation procedure involved the sample carbonization at 700 °C under argon atmosphere. The description of the produced samples is given in Table 1.

The morphology of the obtained materials was studied using a scanning electron microscope (SEM Hitachi SU 8020, Tokyo, Japan). Identification of phases in the produced samples was carried out on the Empyrean PANalytical diffractometer (Malvern Panalytical Ltd., Malvern, UK). The phase composition was determined using the X-ray diffraction method The measurements were performed using Cu Kα radiation. The data were analyzed using the HighScore+ v.4.0 software and the ICDD PDF-4+ database.

The magnetic properties of the samples were studied with the use two magnetom-eter types. At first we measured the magnetic susceptibility at temperatures below room temperature with the use of the LakeShore 7229 susceptometer system. This system allowed us to study the magnetic susceptibility of the samples excited with the use of the alternating magnetic field with amplitude not exceeding 1 mT over temperature range from 4.3 K up to about 320 K. The magnetic susceptibility measurements were supplemented with DC magnetization measurements performed using a Physical Property Measurement System (Quantum Design, San Diego, USA) with the VSM option. The temperature dependences were collected at 50 Oe during heating from 300 K and up to the limit temperature determined by thermogravimetric measurements defining the range of temperatures that do not cause degassing of the sample components. The measurements of the magnetization curves, M(B), were performed at 300 K up to the magnetic field equals to 4 T.

## 3. Results

### 3.1. Structural Characterization

**Figure 1:**
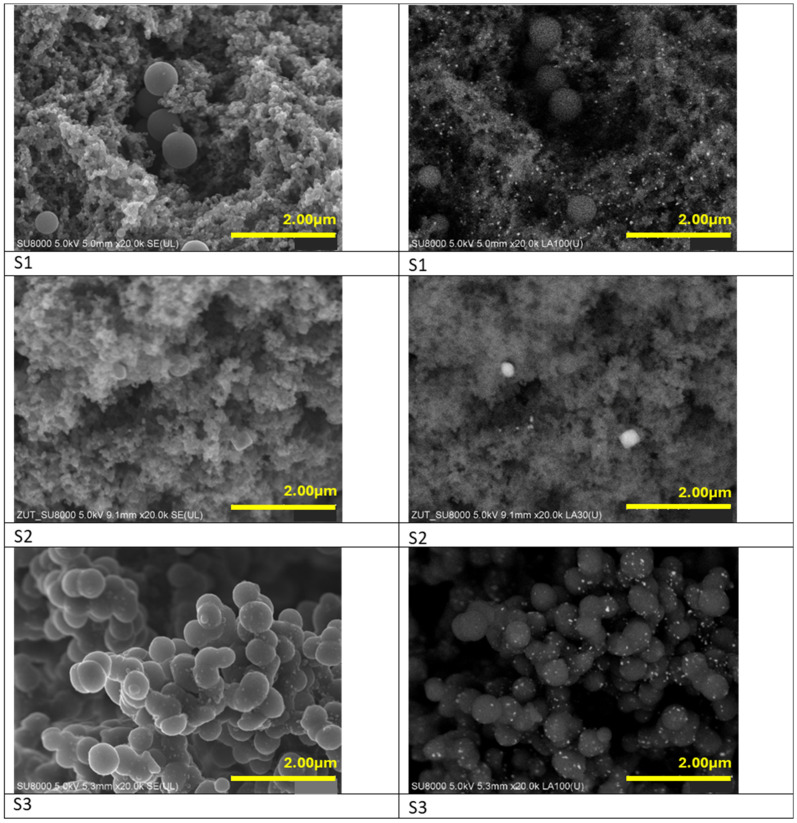
SEM (**left panel**) and BSE (**right panel**) images of the non-activated materials modified with iron nitrate (S1), iron chloride (S2) and iron citrate (S3).

Figure 1 shows SEM images of the non-activated materials (samples S1–S3). Our previous studies [11] proved, that the morphology of the carbon materials obtained from resorcinol-formaldehyde resin consists of tunable spherical-shaped structures. Application of the additional modifier affected the morphology of the resulting materials [12]. Within the present study, we investigated the changes in structure of the carbon material doped with various iron precursors. According to SEM images in Figure 1, the structure of the non-activated material where iron citrate was used as an iron precursor (S3) is composed of clustered, irregular carbon spheres in diameter about 500 nm. Utilization of iron nitrate as an iron precursor resulted in the presence of very low amount of carbon spheres of ca. 400 nm in size among disordered, shapeless carbon structure (S1). When iron chloride was used, no spherical shapes have been obtained (S2). Apparently, the presence of chloride anions inhibits the formation of carbon spheres. The application of iron citrate and iron nitrate provided a very good dispersion of iron particles. In the carbon matrix of S1 and S3 samples the iron particles are evenly distributed reaching mean size below 20 nm. In the case of application of iron chloride (S2), presence of unevenly distributed larger iron particles, about 200 nm in size, have been noticed.

**Figure 2:**
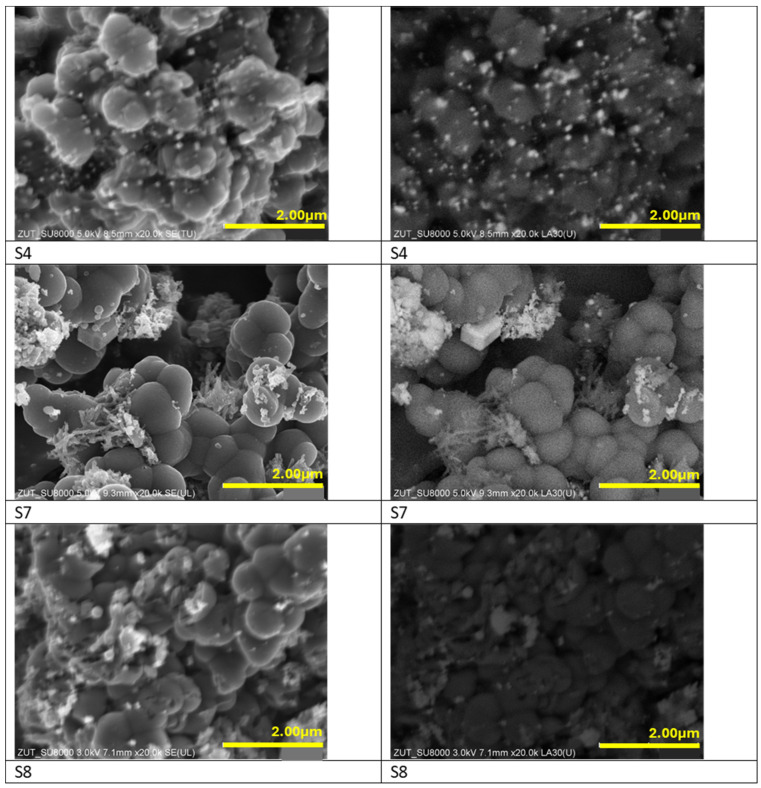
SEM (**left panel**) and BSE (**right panel**) images of the materials activated with potassium oxalate and modified with iron citrate (S4), iron nitrate (S7) and iron chloride (S8).

The morphology of the samples activated with potassium oxalate and modified with iron compounds is presented in Figure 2. When potassium oxalate has been applied, the general spherical shape of the materials has been preserved. Regardless of the used iron compound all materials consisted of clustered carbon spheres ranging about 800 nm in size. In the case of non-activated samples, the use of iron chloride inhibited the formation of carbon spheres, while it was not the case of KOH activated sample doped with iron chloride. The explanation could be a formation of potassium chloride in the latter case, which temperature of decomposition is much higher than that of iron chloride.

The highest level of the dispersion of iron particles among activated materials has been noticed for the sample S4 modified with iron citrate, although the size of iron particles is higher than for non-activated material (S3). Samples modified with iron nitrate and iron chloride were characterized by uneven distribution of iron particles.

**Figure 3:**
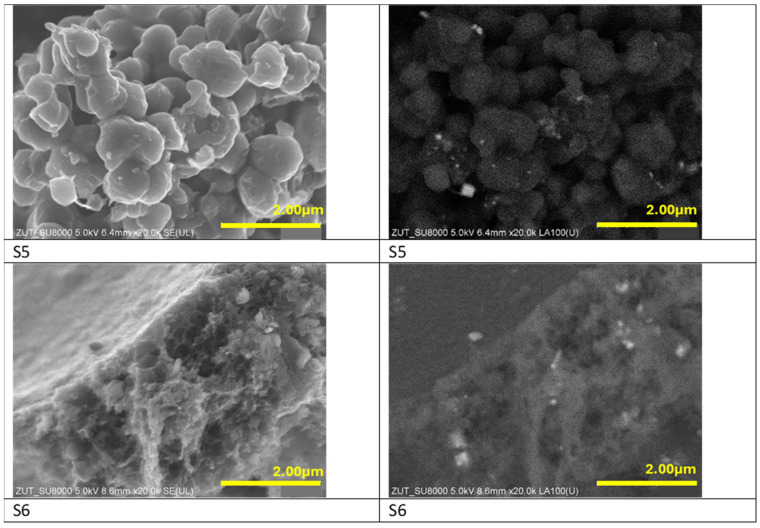
SEM (**left panel**) and BSE (**right panel**) images of the materials activated with potassium hydroxide and modified with iron citrate (S5) and iron chloride (S6).

To compare the influence of different activation methods on the morphology of the samples, two samples have been activated using potassium hydroxide and the SEM images of these materials are presented in Figure 3. The sample S5 modified with iron citrate consists of angular carbon shapes, whereas the sample S6, where iron chloride was used, has a spongy carbon structure. In both cases the iron particles were unevenly distributed.

**Figure 4:**
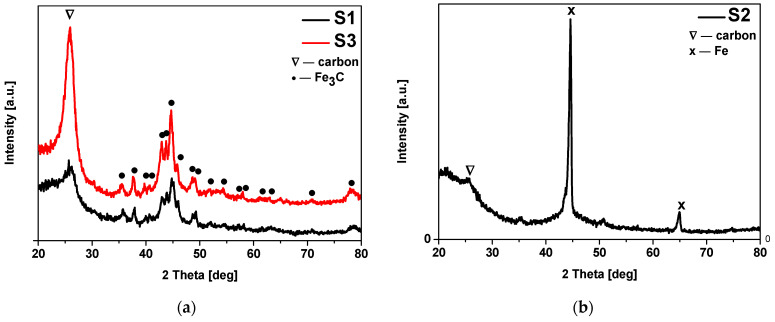
Diffraction patterns of the non-activated materials modified with (**a**) iron nitrate (S1), iron chloride (S2) and (**b**) iron citrate (S3). Reflexes attributed to: Fe_3_C are marked as • (ICDD 00-006-0688), C are marked as ∇ (ICDD 00-041-1487), and Fe are marked as × (ICDD 01-087-0721).

Analysis of the diffraction patterns of non-activated materials modified with iron nitrate (S1) and iron citrate (S3) (Figure 4a) revealed the same phase composition. In both cases, apart from carbon (ICDD 00-041-1487), the peaks corresponding to iron carbide (ICDD 00-006-0688) were identified. The XRD spectra of the non-activated sample modified with iron chloride (S2) (Figure 4b) showed the presence of carbon (ICDD 00-041-1487) and metallic iron (ICDD 01-087-0721). The presence of peaks corresponding to Fe (200) at 2θ = 65 ° and to Fe (110) at 2θ = 44.5 ° was noticed in this sample. In all non-activated samples the diffraction peak corresponding to the graphitic carbon (200) at 2θ = 24° was noticed.

**Figure 5:**
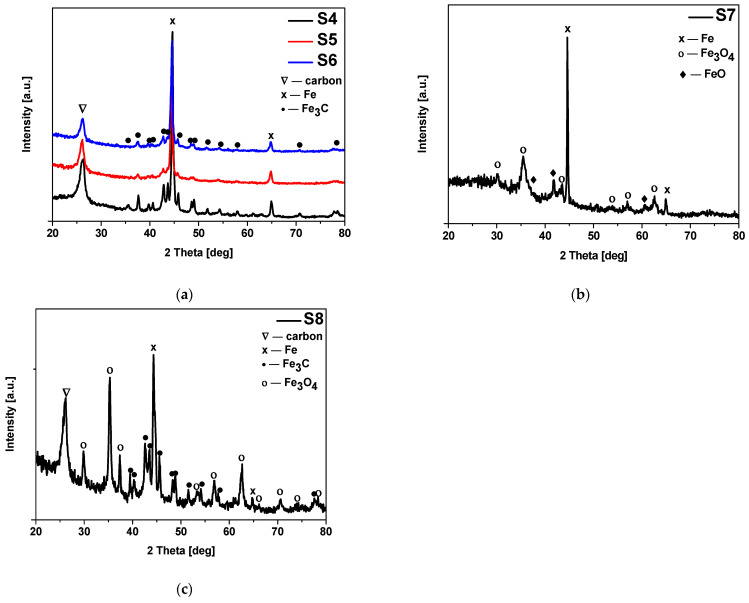
Diffraction patterns of the materials (**a**) activated with potassium oxalate or potassium hydroxide and modified with iron citrate (S4 and S5) and iron chloride (S6); (**b**) activated with potassium oxalate and modified with iron nitrate (S7); (**c**) activated with potassium oxalate and modified with iron chloride (S8). Reflexes attributed to: Fe_3_C are marked as • (ICDD 00-006-0688), C are marked as ∇ (ICDD 00-041-1487), Fe are marked as × (ICDD 01-087-0721), Fe_3_O_4_ are marked as ○ (ICDD 01-087-2334) and FeO are marked as ♦ (ICDD 01-089-0687).

Analysis of the diffraction patterns of the activated materials modified with iron citrate (S4 and S5) and iron chloride (S6) showed the same phase composition of the samples (Figure 5). Potassium oxalate was used as an activator in the sample S4, and in the samples S5 and S6 potassium hydroxide was applied. For all mentioned materials the presence of carbon (ICDD 00-041-1487), iron carbide (ICDD 00-006-0688) and iron (ICDD 01-087-0721) was confirmed.

The XRD spectra of the samples activated with potassium oxalate and modified with iron nitrate (S7) and iron chloride (S8) are shown in Figure 5. In the sample S7 presence of iron (ICDD 01-087-0721), magnetite Fe_3_O_4_ (ICDD 01-087-2334) and ferrous oxide FeO (ICDD 01-089-0687) was confirmed. It should be noted that we didn’t observe presence of a crystalline carbon form in this sample. In the sample S8, like in the case of the S4, S5 and S6 samples, the presence of carbon (ICDD 00-041-1487), iron (ICDD 01-087-0721) and iron carbide (ICDD 00-006-0688) was confirmed. Moreover, we observed the presence of magnetite Fe_3_O_4_ (ICDD 01-087-2334) in this material.

Table 2 shows textural properties of the obtained samples. The surface area values of nonactivated samples ranged from 400 m^2^/g for S3 sample modified with iron citrate to 503 m^2^/g for S2 sample modified with iron chloride. The highest total pore volume value, 0.46 cm^3^/g, has been achieved for the sample S1 modified using iron nitrate. In general, additional chemical activation of samples led to the development of surface area and porosity of the samples. In addition, samples activated using potassium hydroxide expressed higher surface area values and the highest values of specific surface area, 1095 m^2^/g, as well as total pore volume, 0.86 cm^3^/g, were noticed for the sample S6 produced with the use of iron chloride. Also the content of microporosity in this sample was the highest and equaled 0.46 cm^3^/g. Among samples activated using potassium oxalate the highest value of surface area was achieved for S8 sample modified also with iron chloride. Only in once case chemical activation led to the diminishment of surface area and porosity. When iron nitrate and potassium oxalate were used simultaneously, the surface area value and total pore volume value were reduced to 136 m^2^/g and 0.12 cm^3^/g, respectively. 

**Figure 6:**
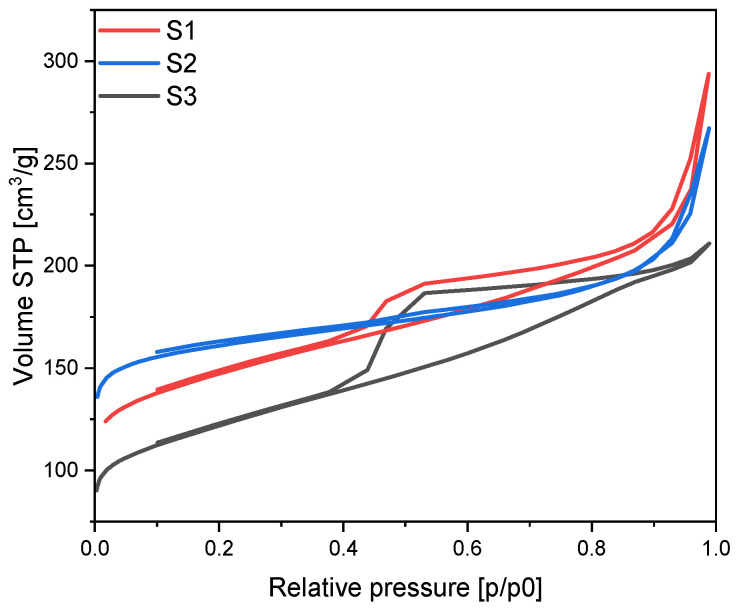
N_2_ sorption isotherms for the nonactivated materials modified using different iron sources.

The nitrogen sorption isotherms for the nonactivated materials are presented in Figure 6. All isotherms are of type II according to the IUPAC classification which is characteristic for macroporous materials. Moreover, for S1 and S3 materials H4 hysteresis loops appeared which is an indication of the filling of micropores in material. This kind of hysteresis loop is characteristic for micro-mesoporous materials [13]. 

**Figure 7:**
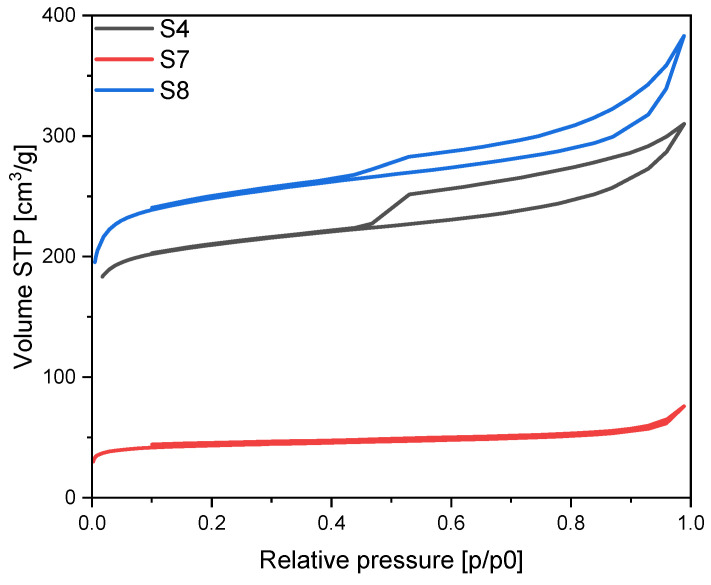
N_2_ sorption isotherms for the potassium oxalate activated materials modified using different iron sources.

The nitrogen adsorption isotherms of the materials activated using potassium oxalate are given in the Figure 7. The lowest amount of adsorbed nitrogen was achieved for the sample modified with iron nitrate, and the shape of the isotherm indicates microporous character of this material (type I). The isotherms obtained for the samples modified with iron chloride and iron citrate are mixed type I and II with H4 hysteresis loops indicating the presence of micro- and macropores in these samples.

**Figure 8:**
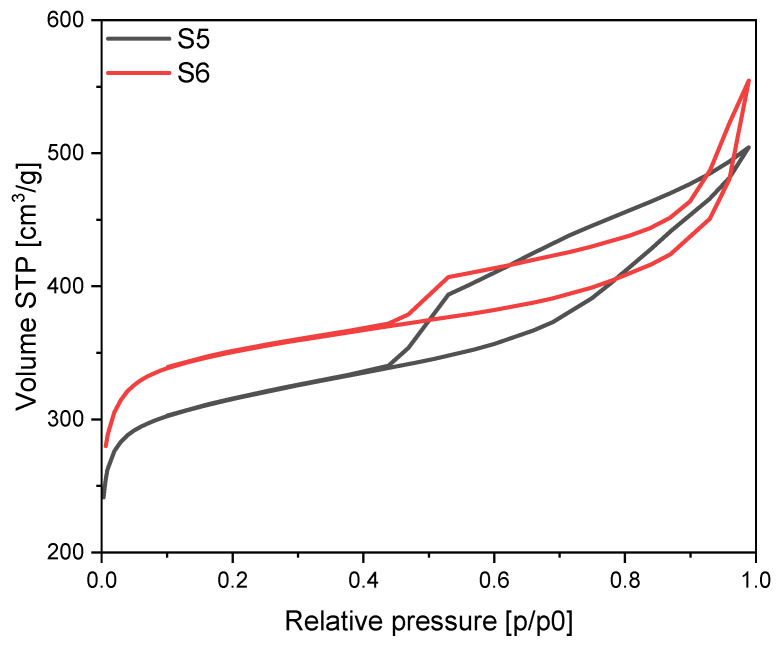
N_2_ sorption isotherms for the potassium hydroxide activated materials modified using different iron sources.

When potassium hydroxide was used as an activator also mixed type I and type II isotherms were noticed (Figure 8). For the S5 material modified using iron citrate H3 hysteresis loop was noticed, which is characteristic for macroporous materials. In addition, for the S6 material, which was modified using iron chloride, H4 hysteresis loop was obtained indicating micro- and mesoporous character of the surface of this material. 

### 3.2. Magnetometric Results

**Figure 9:**
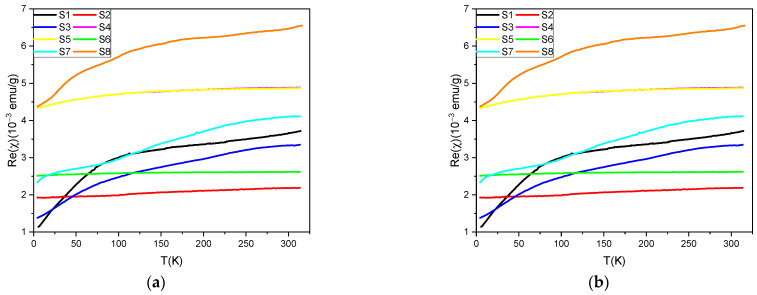
Results of the temperature dependent magnetic measurements for all the samples including: (**a**) real part of the *ac* magnetic susceptibility and (**b**) *dc* magnetization collected at 50 Oe during heating from 300 K.

The results of the temperature dependent magnetometric measurements including: magnetic susceptibility, Re(X(T)), and magnetization, M(T), for all the samples are presented in Figure 9.

As we can clearly see in Figure 9 both the magnetic susceptibility and magnetization vs temperature shows features dominated by the magnetic phases observed via X-ray diffraction characterization. It will be therefore clarified in the following sections after a complete set of magnetometric results will be analyzed.

**Figure 10:**
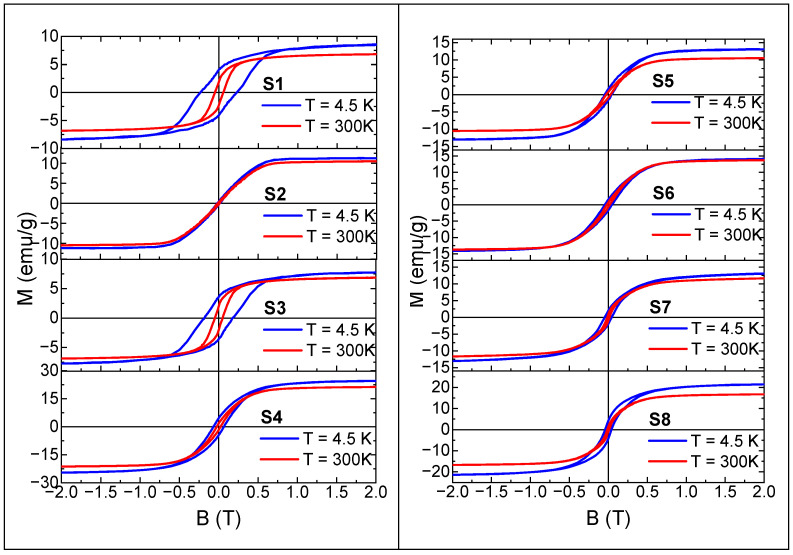
Magnetization as a function of magnetic field obtained at *T* = 4.5 K (blue curves) and at *T* = 300 K (red curves).

Temperature dependent magnetometric measurements were supplemented with the isothermal magnetization as a function of magnetic field measurements. All our measurements were subject to initial data analysis in order to remove contributions from the sample holder. The obtained M vs B dependences are shown in Figure 10. 

All the M(B) curves obtained show a clear ferromagnetic contribution from the iron based magnetic phases detected in the tested samples. The M(B) curves for all samples show saturation of the magnetization and for S1, S2, S3, and S5 samples the appearance of a diamagnetic contribution from carbon present in the samples is visible in high magnetic fields at T = 4.5 K. The value of the magnetization in the M(B) saturation region, named M_S_, observed in the samples varies significantly from about 8 emu/g to 22 emu/g. The magnetic hysteresis loops observed at T = 4.5 K show significant differences in the values of the coercive field, H_C_, remanence magnetization, M_R_, and ellipticity of the hysteresis loop between the samples. Estimated values of Ms, Hc, and M_R_ at both temperatures for all samples are gathered in Table 3. 

## 4. Discussion

Starting from the analysis of the registered Re(X(T)) relations it should be mention that the system consisting of a non-interacting single domain magnetic nano-grains which are well distributed into the nonmagnetic matrix may exhibit so-called blocking process with the temperature decrease. This process is characterized by the blocking temperature T_B_, above which the system is in a superparamagnetic state (exhibits paramagnetic behavior), while below T_B_ is in a block state and non-zero coercivity appears [14]. In the temperature dependence of the AC magnetic susceptibility the blocking process revealed as a well-defined maximum [15]. For Fe_3_C nano-grains embedded into the carbon matrix the literature data indicates a blocking process at low temperatures for which TB decreasing with the increase of the carbon content in the composite [16]. However, a much more important observation resulting from the [16] work is the fact that T_B_ increases with the annealing temperature of the samples changing between 350 °C and 550 °C to such an extent that it exceeds the maximum measurement temperature in the [16] work. As can be seen in Figure 9a, all observed χ(T) dependences for samples in which Fe_3_C nanoparticles were detected, no clear peak in the χ(T) dependence was observed, which would indicate the presence of superparamagnetic blocking. Thus, this is a result analogous to those presented in paper [16] for samples annealed at high temperatures. In view of the above arguments, it should be concluded that the preparation procedures used for the currently tested samples had a similar effect on their magnetic properties as the high-temperature annealing shown in paper [16]. 

The M(T) curves registered for all samples except S2 and S7 show a sharp drop of the magnetization value with increasing temperature from 450 to 500 K, which can be associated with the well-known ferromagnetic-paramagnetic phase transition of cementite. The Curie temperature, T_C_, for bulk Fe_3_C reported in the literature is between 483 K and 488 K [17,18], and here T_C_ defined as the maximum in the first derivative of the M(T) curves is ~480 K for all samples containing this phase. Registered lowering of the T_C_ value most probably becomes from the scaling down of Fe_3_C from bulk to the nano-grains [19]. For temperatures higher then T_C_ of cementite, the M(T) dependences for samples S1, S3–S6, S8 indicates other features, which should be related to the presence of the other magnetic phases. However these additional phases cannot be clearly identified because they may result from conversion between phases during the measurement with heating [20]. Taking a holistic look at the shape of the M(T) relations, the relationship with the sample composition is observed, i.e., similar shape of the M(T) curves have samples S1 and S3, and also S4, S5, S6 together. It should be also stressed that for all samples besides S1 and S3 the magnetic contribution from the α-Fe phase (T_C_ = 1044 K) [21] is responsible for the background of the M(T) relations. In the case of the S2 sample, the M(T) relation does not revealed any anomalies similar to these observed for the other samples. This is consistent with the XRD measurements for S2, in which the only recognized magnetic phase is α-Fe. 

Structural characterization of the samples showed that in two of them, S7 and S8, the presence of the Fe_3_O_4_ phase was detected. Characteristic low-temperature phase transition for Fe_3_O_4_ is the Verwey transition which in the case of volumetric samples occurs at T~125 K, and manifests as a sharp drop of the magnetization value with the temperature decrease [22]. In the Re(X(T)) dependences for S7 and S8 it is hard to recognize any feature, like step, in this region, however this does not indicate a lack of presence of Fe_3_O_4_. Apart from the above, Fe_3_O_4_ shows a ferrimagnetic spinel phase with a Curie temperature of 858 K [23]. The M(T) results obtained for the above two samples, unfortunately, do not allow to clearly indicate the phase transitions related to the magnetite phase, due to temperature limit of the measurements.

Table 3 summarizes several parameters determined on the basis of the measurement results of the M(B) curves collected in Figure 10. At the begging it should be stressed that the estimates M_S_ represents the magnetization value of the sample in the saturation region. Due to the multiphase nature of the investigated samples, the estimated M_S_ values cannot be directly compared with the saturation magnetization values of the magnetic phases included in the samples. However, all M_S_ listed in Table 3 are lower than the bulk values for all identified magnetic phases in all samples, which confirmed the presence of a nonmagnetic phase, i.e., carbon. Both M_S_ and H_C_ show a typical increase in their values with decreasing temperature. For the S1 and S3 samples, for which the only recognized magnetic phase in the XRD measurements is the Fe_3_C, there is a good accordance between the parameters included in Table 3 and an analogy in the shape of the M(B) curves. Taking into account above-mentioned similarity of the M(T) relations for these two samples, it can be concluded that despite the use of a different iron precursor in the synthesis, magnetically similar systems were obtained. According to the M(B) relations for S2 it may be also concluded that this sample exhibit the behavior of the soft magnetic material typical for α-Fe, which coincide with the phase composition. For samples S4, S5, and S6 which contain the same magnetic phases, i.e., Fe_3_C and Fe, M_S_ and H_C_ differs between each other, and also the M(B) shapes do not coincides. The reasons for these discrepancies can be found in the composition, internal magnetic and structural parameters of S4, S5, and S6, which is primarily related to different synthesis methods. The M(B) measurement results obtained for S7 and S8, are hard to compare with other samples because they differ in phase composition from the other samples and also from each other. Summarizing, the results of the magnetic measurements clearly indicate the formation of magnetic phases in the synthesis methods used. However, the multiphase character of the samples complicates drawing unambiguous conclusions about the magnetic properties of the precipitated in the carbon matrix nanoparticles.

## 5. Conclusions

A series of magnetic composites containing various iron compounds and highly microporous carbon spheres was obtained using a modified Stöber method in the microwave assisted solvothermal reactor. Resorcinol and formaldehyde were used as carbon precursors and iron citrate, nitrate and chloride were used as a source of iron in the composites. Some samples were additionally activated with potassium compounds. The produced samples contained highly porous carbon and iron, iron carbide or magnetite. As the coupling of the high porosity of carbon spheres with magnetic properties of iron compounds provides a potential application of the composites to removal of impurities in water, followed by a magnetic separation of the sorbent, a particular attention has been paid to magnetic properties of the produced materials. All of them have magnetic properties enabling their magnetic separation, however the non-activated samples containing cementite (Fe_3_C) are characterized by the most significant H_C,_ M_S_ and M_R_ values.

## Figures and Tables

**Table 1:** Description of the iron/carbon spheres composites.

No.	Iron Precursor	Activator
S1	Fe(NO_3_)_3_·9H_2_O	No
S2	FeCl_3_·6H_2_O	No
S3	C_6_H_5_O_7_Fe	No
S4	C_6_H_5_O_7_Fe	K_2_C_2_O_4_·H_2_O
S5	C_6_H_5_O_7_Fe	KOH
S6	FeCl_3_·6H_2_O	KOH
S7	Fe(NO_3_)_3_·9H_2_O	K_2_C_2_O_4_·H_2_O
S8	FeCl_3_·6H_2_O	K_2_C_2_O_4_·H_2_O

**Table 2:** Textural properties of the obtained materials.

No.	S_BET_ [m^2^/g]	TPV [cm^3^/g]	Vmic [cm^3^/g]
S1	476	0.46	0.18
S2	503	0.41	0.22
S3	400	0.33	0.13
S4	656	0.48	0.27
S5	991	0.78	0.39
S6	1095	0.86	0.46
S7	136	0.12	0.06
S8	780	0.60	0.33

S_BET_—specific surface area; TPV—total pore volume; Vmic—volume of micropores <2 nm.

**Table 3:** Selected parameters related to magnetic properties of the samples studied within this work including the coercive field, H_C_, (obtained at T = 4.5 K and at room temperature) saturation magnetization, M_S_, and remanence magnetization, M_R_, to M_S_ ratio.

No.	Phases	M_S_ [emu/g] 4.5 K/300 K	H_C_ [mT] 4.5 K/300 K	M_R_ [emu/g] 4.5 K
S1	Fe_3_C	8.5/6.8	233/49	4.0
S2	Fe	11.2/10.8	16/8	0.38
S3	Fe_3_C	7.8/6.8	183/44	3.6
S4	Fe_3_C, Fe	24.8/21.3	66/27	4.5
S5	Fe_3_C, Fe	13.2/10.5	44/26	1.6
S6	Fe_3_C, Fe	14.2/13.7	46/20	1.6
S7	Fe, Fe_3_O_4_, FeO	14.6/12.9	46/10	1.9
S8	Fe_3_C, Fe, Fe_3_O_4_	21.9/17.7	42/12	4.1

## Data Availability

The data presented in this study are available upon request from the corresponding author.
